# Supplementary material for: What does it take to learn the rules of RNA base pairing? A lot less than you may think
Source: bioRxiv. 2025 Aug 2:2025.07.31.668042. Preprint. [Version 1] doi: 10.1101/2025.07.31.668042 (PMC12324431; doi:10.1101/2025.07.31.668042)
Supplement: Supplement 1 [file media-1.gz › supplemental_material/src/rscape_v2.6.0.tar.gz!/rscape_v2.6.0/lib/infernal/Manuscripts/filter_thresholds/figs/mervtime.pdf]

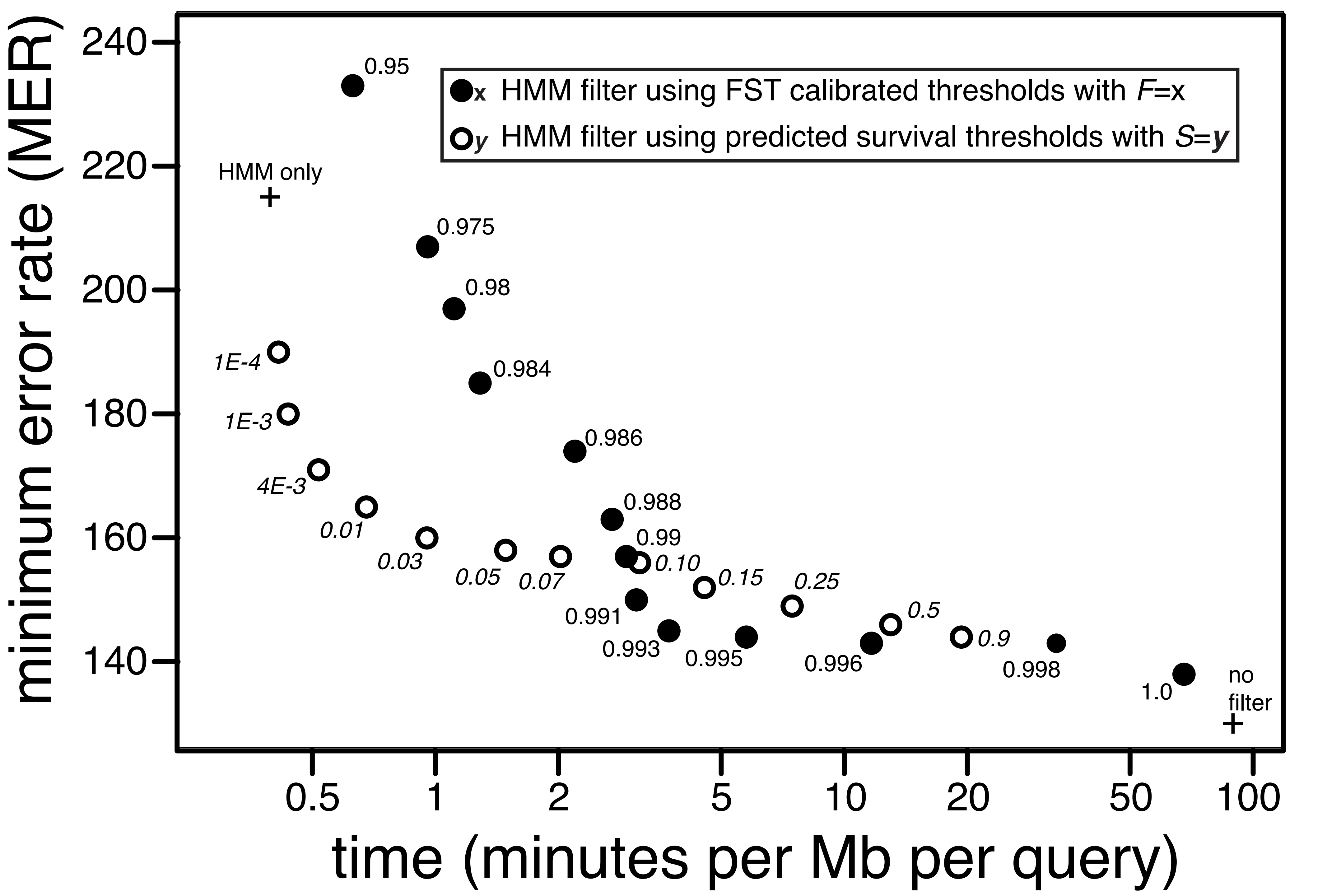

HMM filter using FST calibrated thresholds with F=x
HMM filter using predicted survival thresholds with S=y
HMM only
no filter
0.95
0.975
0.98
0.984
0.986
0.988
0.99
0.991
0.993
0.995
0.996
0.998
1.0
1E-4
1E-3
4E-3
0.01
0.03
0.05
0.07
0.10
0.15
0.25
0.5
0.9
240
220
200
180
160
140
minimum error rate (MER)
0.5
1
2
5
10
20
50
100
time (minutes per Mb per query)
